# Antibodies against lytic and latent Kaposi's sarcoma-associated herpes virus antigens and lymphoma in the European EpiLymph case–control study

**DOI:** 10.1038/bjc.2011.392

**Published:** 2011-09-27

**Authors:** Y Benavente, G Mbisa, N Labo, D Casabonne, N Becker, M Maynadie, L Foretova, P L Cocco, A Nieters, A Staines, P Bofetta, P Brennan, D Whitby, S de Sanjosé

**Affiliations:** 1 Unit of Infections and Cancer (UNIC), Cancer Epidemiology Research Programme, Institut Català d’ Oncologia, IDIBELL, Gran Via km 199-203, 08908L’ Hospitalet de Llobregat, Barcelona, Spain; 2 CIBER de Epidemiología y Salud Pública (CIBERESP), Spain; 3 Viral Technology Laboratory, Advanced Technology Program, SAIC-Frederick, Inc., NCI-Frederick, Frederick, MD 21702, USA; 4 Viral Oncology Section, AIDS and Cancer Virus Program, SAIC-Frederick, Inc., NCI-Frederick, Frederick, MD 21702, USA; 5 Division of Cancer Epidemiology, German Cancer Research Center, Im Neuenheimer Feld 280, D-69120 Heidelberg, Germany; 6 Registre des Hémopathies Malignes de Côte d’Or, EA 4184, University of Burgundy; Biological Hematology Unit; CRB Ferdinand Cabanne, Universitary Hospital of Dijon, Dijon, France; 7 Cancer Epidemiology & Genetics, Masaryk Memorial Cancer Institute, Brno, Czech Republic; 8 Department of Public Health, Occupational Health Section, University of Cagliari, Cagliari, Italy; 9 Department of Molecular Epidemiology, Centre of Chronic Immunodeficiency, University Medical Centre Freiburg, Freiburg i. Br., Germany; 10 Department of Molecular Tumour Epidemiology, German Cancer Research Center, Heidelberg, Germany; 11 Public Health University College, Dublin, Ireland; 12 Tisch Cancer Institute, Mount Sinai School of Medicine, New York, NY, USA; 13 International Prevention Research Institute, Lyon, France; 14 IARC, International Prevention Research Institute, 95 cours Lafayette, 69006 Lyon, France

**Keywords:** epidemiology, Kaposi's sarcoma-associated herpes virus, human herpes virus 8, serology, lymphoma

## Abstract

**Background:**

Kaposi's sarcoma-associated herpes virus is associated with primary effusion lymphoma and multicentric Castleman's disease.

**Methods:**

Seropositivity to lytic and latent Kaposi's sarcoma herpes virus (KSHV) antigens were examined in 2083 lymphomas and 2013 controls from six European countries.

**Results:**

Antibodies against KSHV latent and lytic antigens were detectable in 4.5% and 3.4% of controls, respectively, and 3.6% of cases (*P*>0.05). The KSHV seropositivity was associated with splenic marginal zone lymphoma (SMZL) (odds ratio (OR)=4.11, 95% confidence interval (CI)=1.57–10.83) and multiple myeloma (OR=0.31, 95% CI=0.11–0.85).

**Conclusion:**

The KSHV is unlikely to contribute importantly to lymphomagenesis among immunocompetent subjects. However, the observed association with SMZL may underline a chronic antigen mechanism in its aetiology.

Kaposi's sarcoma herpes virus (KSHV) also known as human herpes virus-8 is tightly associated with the development of Kaposi sarcoma (KS) and primary effusion lymphoma (PEL), and there is limited evidence of an association with multicentric Castleman's disease (MCD) (Bouvard *et al*, 2009). These malignancies occur more frequently in immunosupressed patients, particularly in human immunodeficiency virus (HIV)-infected subjects.

Literature is scarce in relation between KSHV infection and the development of non-Hodgkin lymphoma (NHL). In 2004, a case–control study carried out among immunocompetent patients in Spain failed to identify a major contribution of KSHV infection to lymphomagenesis. Although based on few cases, KSHV seropositive patients were more likely to have lymphoplasmacytic lymphoma and low-grade lymphoma (de Sanjose *et al*, 2004). A prospective study in the United Kingdom revealed that KSHV lytic and latent antibodies did not appear to be associated with NHL in HIV-infected individuals (Newton *et al*, 2006).

The aim of this study was to investigate the association between KSHV seropositivity and lymphoma subtypes in the multicenter European case–control study EpiLymph.

## Materials and methods

### Study population

Information was collected on 2362 incident lymphoma cases and 2465 controls during 1998–2003 in six countries (Germany, Italy, Spain, Ireland, France and Czech Republic). In Germany and Italy, population-based controls were sampled whereas, in all the other countries, hospital-based controls were recruited. The participation rate was 68.5% and 87.7% among controls and cases, respectively. The study has been described elsewhere (de Sanjose *et al*, 2006). All subjects signed an informed consent, and local ethics review committees approved the study. The KSHV serology was performed on 84% (*n*=2118) of cases and 82% (*n*=2048) of controls. After excluding immunosupressed patients, 2083 cases and 2013 controls were included in this study.

### KSHV serology

All serum samples were tested for antibodies to KSHV lytic and latent antigens. Laboratory personnel performing the assay were blinded to patient disease status and demographic characteristics of the subjects. Details of the testing procedure are described elsewhere (Mbisa *et al*, 2010). Shortly, antibodies against the lytic antigen K8.1 and latent antigen LANA encoded by ORF73 were measured using an enzyme-linked immunosorbent assay (ELISA) based on recombinant proteins. A small set of samples from KS patients and a panel of 100 blood donor samples were run as ELISA positive and negative controls, respectively. OD cutoffs for seropositivity for each plate were defined as the average of negative controls plus 0.75 for the K8.1 ELISA and the average of the negative controls plus 0.35 for the ORF73 ELISA, to account for plate-to-plate variability. In validation studies using US blood donors and AIDS KS patients, the K8.1 assay has shown 98.78% sensitivity and 98.79% specificity, while the ORF73 assay had 89.02% sensitivity and 97.57% specificity (Mbisa *et al*, 2010).

### Statistical analysis

Differences in KSHV seropositivity by categorical variables were done by means of a *χ*^2^-test. Unconditional logistic regression was used to estimate the odds ratios (ORs) and 95% confidence interval (95% CI) of lymphoma in relation to KSHV seropositivity. All models were adjusted for age, sex and country. Two-sided *P*-values were considered statistically significant at the 0.05 level. Heterogeneity among countries was evaluated with a likelihood ratio test comparing the model with and without interaction between country and exposure. We used the kappa (*κ*) statistic to describe the agreement between assays and the results of the two assays.

We carried out sensitivity analyses to evaluate the influence of country, type of control, cutoffs for seropositivity and age on our results, and to reduce the possibility of misclassification of the KSHV status. All the analyses were conducted using STATA (version 10.1, College Station, TX, USA) and R (version 2.10.1, 2009, The R Foundation for Statistical Computing, ISBN 3-900051-07-0).

## Results

Table 1 shows the distribution of cases and controls and KSHV seroprevalence among controls according to demographic characteristics. The average age at entry was 56.4 (s.d.: 16.1) for cases and 56.2 for controls (s.d.: 16.4). Among controls, seropositivity was 4.5% for ORF73 (90 out of 2013) and 3.4% for K8.1 (68 out of 2013). The prevalence of anti-KSHV antibodies did not differ significantly by sex (*P*=0.5), increased linearly with age (*P*-trend <0.0001) and decreased with increasing educational level (*P*-trend=0.03) for latent antibodies. The highest seroprevalence was observed in Italy (12%). Similar pattern for K8.1 antibodies was observed in relation to demographic characteristics. Seropositivity among controls for both antibodies represented 2% (*n*=38). Overall, the pairwise agreement between ORF73 and K8.1 results among controls was moderate (*κ*=0.46), being good in the youngest (*κ*=0.61) and in the age group of 64 to 70 years old (*κ*=0.63) and in Italy (*κ*=0.74) (data not shown).

Table 2 shows the OR by lymphoma subtypes in relation to anti-KSHV antibody status. For all lymphoma, we observed no association with seropositivity to ORF73 (OR=0.78, 95% CI=0.57–1.07, seropositive cases/total cases *N*=75/2083) nor to K8.1 (OR=1.05, 95% CI=0.76–1.48, *N*=74/2083). By lymphoma subtype, CLL/SLL and MM cases were more likely to be seronegative to latent anti-KSHV antibodies than controls (OR=0.58, 95% CI=0.31–1.06, *N*=13/375 and OR=0.31, 95% CI=0.11–0.85, *N*=4/254, respectively). Antibodies against K8.1 antigen, but not for ORF73 antigen, showed a significant increased OR for splenic marginal zone lymphoma (SMZL) (OR=4.11, 95% CI=1.57–10.83, *N*=6/36). Although 70% (*N*=25) of SMZL cases came from Spain, sensitivity analyses on country did not modify the results (data not shown). We also observed a nonsignificant increased OR for mantle cell lymphoma (MCL) (OR=2.63, 95% CI=0.88–7.86, *N*=4/58). Further sensitivity analyses on age, type of control and OD cutoff variations did not change the results.

## Discussion

This study analysed the association between antibodies against KSHV and lymphoma in the European case–control study EpiLymph. Overall, we found no association between positivity to lytic or latent antibodies against KSHV antigens and lymphoma. However, we observed an increased OR in SMZL and MCL, and a decreased OR in MM. Furthermore, our data support previous observations in which seropositivity to KSHV increases with increasing age and is higher in Italy than in other European countries (Whitby *et al*, 1998).

We observed that KSHV seroreactivity to lytic antigens increased four times the probability of SMZL. This was consistent with our previous results based on Spanish cases (de Sanjose *et al*, 2004). This analysis adds 11 further SMZL cases from 4 other countries reinforcing the association. The SMZL is a low-grade B-cell lymphoma belonging to the marginal zone lymphoma group that involves predominantly the spleen. There is amounting evidence that SMZL may be related to chronic antigen immune stimulation, in particular to chronic hepatitis C (HCV) (Suarez *et al*, 2006). The mechanism by which KSHV could induce SMZL is unclear. However, KSHV is a lymphotropic virus that can produce PEL, a large B-cell lymphoma arising in KSHV-associated MCD, and an entity named germinotropic lymphoproliferative disorder that has been described in the absence of HIV (Carbone *et al*, 2005). In addition, malignant cells from SMZL may be affected by microbial pathogens because they arise from marginal zone cells that are in the underlying pathways of the immune response to infection (Suarez *et al*, 2006). Further, KSHV is the aetiological trigger of KS in the elderly in the absence of HIV or HCV. All seropositive subjects in our analysis were HCV and HIV negative.

In our data, we detected a nonsignificant increased OR in MCL. To our knowledge, this has not been previously reported and we do not have a clear biological explanation. The MCL may be subject to histological classification (Weisenburger and Armitage, 1996). However, EpiLymph cases were reviewed by an international panel of pathologists, ruling out major misclassifications by lymphoma subtypes. Further studies should be relevant to help in the understanding of this association.

The observed reverse association of KSHV and MM is consistent with the literature (Rettig *et al*, 1997). We had previously reported within EpiLymph, lower antibody response to Helicobacter Pylori, simian virus 40 and Epstein-Barr virus (de Sanjose *et al*, 2007) in MM subjects compared with controls.

To our knowledge, this is the first study that evaluates the association between anti-KSHV antibody status and lymphoma among immunocompetent European subjects. We have enlarged the sample size of our previous study, including participants from four European countries to validate our previous finding and also to explore further the association with specific lymphoma subtypes. Sensitivity analyses, including variation of seropositivity cutoffs and age-matched analyses, supported that our results were not materially influenced by potential confounding factors. Serological evaluation of KSHV has not been standardised (Pellett *et al*, 2003) but the highest seroprevalence observed in Italy and the increasing antibody titres with age reinforce the validity of the assays. Besides, available data for KSHV prevalence of EpiLymph countries are comparable with our data (Whitby *et al*, 1998). Estimates from hospital-based case–control studies might be biased if the underlying KSHV prevalence in the control population affects hospitalisation rates owing to KSHV-related diseases. However, the results did not change materially after restricting the analyses to hospital or population case–control studies. We cannot also rule out that our results may be due to chance originating from the small KSHV seroprevalence by subtype.

In conclusion, our findings support an association with SMZL among immunocompetent subjects that deserves further research.

## Figures and Tables

**Table 1:** Distribution of cases, controls and seropositivity of KSHV among controls by demographic characteristics

	**Controls**			**Cases**
		**Seropositivity to**		
	***n* (%)**	**ORF73 *n* (%)**	**K8.1 *n* (%)**	***n* (%)**
Number of participants	2013	90 (4.5)	68 (3.4)	2083
				
*Gender*				
Male	1081 (53.6)	50 (4.6)	35 (3.2)	1165 (50.4)
Female	932 (46.4)	40 (4.3)	33 (3.5)	918 (49.6)
				
*Age*				
<40	379 (18.8)	7 (1.8)	6 (1.6)	373 (17.9)
41–54	395 (19.6)	10 (2.5)	7 (1.8)	399 (19.2)
55–63	419 (20.8)	20 (4.8)	14 (3.3)	468 (22.4)
64–70	396 (19.7)	23 (5.8)	17 (4.3)	420 (20.2)
>70	424 (21.1)	30 (7.1)	24 (5.7)	422 (20.3)
				
*Educational level*				
Low	930 (46.2)	48 (5.2)	48 (5.2)	942 (45.2)
Medium	817 (40.6)	34 (4.3)	10 (1.2)	837 (40.2)
High	265 (13.2)	7 (2.6)	10 (3.8)	285 (13.7)
				
*Center of recruitment*				
Hospital-based	1208 (60.0)	54 (4.5)	40 (3.3)	1212 (58.2)
Spain	594 (54.4)	29 (4.9)	30 (5.1)	498 (45.6)
France	174 (38.7)	6 (3.5)	2 (1.2)	276 (61.3)
Ireland	139 (48.4)	3 (2.2)	4 (2.9)	148 (51.6)
Czech Republic	301 (50.9)	16 (5.3)	4 (1.3)	290 (49.1)
Population-based	805 (40.0)	36 (4.5)	28 (3.5)	871 (41.8)
Germany	650 (49.4)	17 (2.6)	13 (2.0)	666 (50.6)
Italy	155 (43.1)	19 (12.3)	15 (9.7)	205 (56.9)

Abbreviations: n=number; KSHV=Kaposi's sarcoma herpes virus.

Numbers may not add due to missing data.

**Table 2:** Adjusted OR and 95% CI for antibodies for latent and lytic KSHV antigens by lymphoma subtypes

		**ORF73**			**K8.1**		
	***N***	***N* (%)**	**OR (95% CI)^a^**	***P***	***N* (%)**	**OR (95% CI)^a^**	***P***
Control	2013	90 (4.5)	Ref		68 (3.4)	Ref	
							
All lymphoma	2083	75 (3.6)	0.78 (0.57–1.07)	0.1	74 (3.6)	1.05 (0.76–1.48)	0.8
							
*B-cell lymphoma*	1660	60 (4.3)	0.76 (0.54–1.06)	0.1	54 (4.0)	1.04 (0.73–1.48)	0.8
Diffuse large B-cell lymphoma	473	23 (4.9)	0.95 (0.59–1.55)	0.8	17 (3.6)	0.96 (0.55–1.68)	0.9
CLL/SLL	375	13 (3.5)	0.58 (0.31–1.06)	0.08	12 (3.2)	0.75 (0.4–1.43)	0.4
Multiple myeloma	254	4 (1.6)	0.31 (0.11–0.85)	0.02	4 (1.6)	0.43 (0.15–1.2)	0.1
Follicular lymphoma	230	10 (4.3)	0.99 (0.49–1.97)	1.0	10 (4.3)	1.43 (0.71–2.9)	0.3
Mucosa-associated lymphoid tissue	84	3 (3.6)	0.74 (0.23–2.43)	0.6	2 (2.4)	0.62 (0.15–2.64)	0.5
B NHL NOS^b^	76	1 (1.3)	0.26 (0.03–1.92)	0.2	4 (5.3)	1.75 (0.59–5.17)	0.3
Mantle cell lymphoma	58	3 (5.2)	1.13 (0.34–3.78)	0.2	4 (6.9)	2.63 (0.88–7.86)	0.08
Lymphoplasmacytic lymphoma	39	4 (10.3)	1.70 (0.56–5.13)	0.3	3 (7.7)	1.55 (0.44–5.42)	0.5
Splenic marginal zone lymphoma	36	2 (5.6)	1.00 (0.23–4.36)	1	6 (16.7)	4.11 (1.57–10.83)	0.004
Hairy cell leukemia	13	—	—	—	1 (7.8)	3.50 (0.41–29.78)	0.3
Precursor B NHL	22	1 (4.5)	2.20 (0.27–17.74)	0.5	1 (4.5)	2.47 (0.3–20.12)	0.4
Hairy cell leukemia	13	—	—	—	1 (7.8)	3.50 (0.41–29.78)	0.3
							
*Hodgkin lymphoma*	303	9 (3.0)	1.04 (0.49–2.21)	0.9	6 (2.0)	0.93 (0.37–2.33)	0.9
Classical Hodgkin lymphoma^c^	284	9 (3.2)	1.14 (0.53–2.44)	0.7	6 (2.1)	1.02 (0.4–2.56)	1.0
Non-classical Hodgkin lymphoma	19	0 (0.0)	—	—	0 (0.0)		
							
*T-Cell lymphoma*	120	2 (1.7)	0.38 (0.09–1.59)	0.2	4 (3.3)	1.06 (0.37–3.01)	0.9
Other T NHL^d^	78	2 (2.6)	0.61 (0.15–2.56)	0.5	3 (3.8)	1.30 (0.39–4.29)	0.7
Mycosis T NHL	29	0 (0.0)	—	—	1 (3.4)	0.74 (0.09–5.71)	0.8
Precursor T NHL	5	0 (0.0)	—	—	0 (0.0)	—	—
Cutaneous T NHL	2	0 (0.0)	—	—	0 (0.0)	—	—
							
NHL nos	6	0 (0.0)	—	—	0 (0.0)	—	—

Abbreviations: OR=odds ratio; CI=confidence interval; N=number; CLL/SLL=chronic lymphocytic leukaemia/small lymphocytic leukaemia; NHL=non-Hodgkin lymphoma.

a Using logistic regression adjusted for age (quintiles), sex and centre of recruitment.

b B NHL NOS: malignant lymphoma, non-Hodgkin (*n*=76).

c Classical HL: classical Hodgkin lymphoma (*n*=37); mixed cellularity classical HL (*n*=65); nodular sclerosis classical HL (*n*=169); lymphocyte-rich classical HL (*n*=9); lymphocyte-depleted classical HL (*n*=4).

d Other T NHL: mature T-cell lymphoma, NOS (*n*=34), angioimmunoblastic T-cell lymphoma (*n*=10), anaplastic large cell lymphoma, T-cell and null cell type (*n*=19), Hepatosplenic gamma-delta cell lymphoma (*n*=2), NK/T-cell lymphoma, nasal and nasal-type (*n*=7), prolymphocytic leukaemia, T-cell type (*n*=1).

Intestinal T-cell lymphoma (*n*=1), T-cell large granular lymphocytic leukemia (*n*=4).
